# Bradykinin Type 1 Receptor – Inducible Nitric Oxide Synthase: A New Axis Implicated in Diabetic Retinopathy

**DOI:** 10.3389/fphar.2019.00300

**Published:** 2019-03-29

**Authors:** Rahmeh Othman, Elvire Vaucher, Réjean Couture

**Affiliations:** ^1^School of Optometry, University of Montreal, Montreal, QC, Canada; ^2^Department of Pharmacology and Physiology, University of Montreal, Montreal, QC, Canada

**Keywords:** diabetic retinopathy, bradykinin type 1 receptor, inducible nitric oxide synthase, kallikrein-kinin system, inflammation, oxidative stress

## Abstract

Compelling evidence suggests a role for the inducible nitric oxide synthase, iNOS, and the bradykinin type 1 receptor (B1R) in diabetic retinopathy, including a possible control of the expression and activity of iNOS by B1R. In diabetic retina, both iNOS and B1R contribute to inflammation, oxidative stress, and vascular dysfunction. The present study investigated whether inhibition of iNOS has any impact on inflammatory/oxidative stress markers and on the B1R-iNOS expression, distribution, and action in a model of type I diabetes. Diabetes was induced in 6-week-old Wistar rats by streptozotocin (65 mg.kg^-1^, i.p.). The selective iNOS inhibitor 1400W (150 μg.10 μl^-1^) was administered twice a day by eye-drops during the second week of diabetes. The retinae were collected 2 weeks after diabetes induction to assess the protein and gene expression of markers by Western blot and qRT-PCR, the distribution of iNOS and B1R by fluorescence immunocytochemistry, and the vascular permeability by the Evans Blue dye technique. Diabetic retinae showed enhanced expression of iNOS, B1R, carboxypeptidase M (involved in the biosynthesis of B1R agonists), IL-1β, TNF-α, vascular endothelium growth factor A (VEGF-A) and its receptor, VEGF-R2, nitrosylated proteins and increased vascular permeability. All those changes were reversed by treatment with 1400W. Moreover, the additional increase in vascular permeability in diabetic retina induced by intravitreal injection of R-838, a B1R agonist, was also prevented by 1400W. Immunofluorescence staining highlighted strong colocalization of iNOS and B1R in several layers of the diabetic retina, which was prevented by 1400W. This study suggests a critical role for iNOS and B1R in the early stage of diabetic retinopathy. B1R and iNOS appear to partake in a mutual auto-induction and amplification loop to enhance nitrogen species formation and inflammation in diabetic retina. Hence, B1R-iNOS axis deserves closer scrutiny in targeting diabetic retinopathy.

## Introduction

DR is a major complication of diabetes mellitus and the leading cause of blindness in working-age adults (Fong et al., 2004). Most patients with type I diabetes develop retinopathy in the first two decades of the disease (Fong et al., 2004). In its early stage, DR is asymptomatic even though the development of vascular bed damage involving changes in retinal vessel diameter, alterations in retinal hemodynamics, increase in leakage of the blood-retinal barrier, overexpression, and accumulation of vascular endothelial growth factor, VEGF (Cheung et al., 2010). This vascular damage progresses into retinal hemorrhage, hard exudates and the appearance of microaneurysms.

Current therapeutic interventions are used only for the late stage of DR when microvascular complications have already resulted in irreversible structural changes. These treatments, including the intravitreal injections of anti-VEGF and laser photocoagulation, are invasive, need to be repeated regularly and are only able to stop the progression of DR without restoring the loss of vision (Fong et al., 2004). Therefore, more effective treatments are needed to intervene in an early stage of DR. Many studies have shown an implication of several pro-inflammatory and oxidative stress mediators in the early stage of this ocular disease (Bhat et al., 2014). Among them, the inducible isoform of NO synthase (iNOS) is overexpressed and activated in ischemic and diabetic rodent retinae (Carmo et al., 2000; Sennlaub et al., 2001, 2002; Ellis et al., 2002; Yuasa et al., 2008; Rosales et al., 2010; Silva et al., 2010) and in diabetic human retina (Abu El-Asrar et al., 2001, 2004). NO is a potent vasodilatory gas and is considered as a free radical due to the presence of an unpaired electron in its valence shell. Being highly reactive, especially with the superoxide anions, NO largely contributes to the oxidative stress, inducing retinal cells damage. Thus, compelling evidence suggests that iNOS is implicated in the onset of DR (Zheng et al., 2007; Mishra and Newman, 2010).

Under physiological situation, NO produced at low amounts by the constitutive isoforms of NOS, endothelial NOS (eNOS) and neuronal NOS (nNOS), plays, however, an important role in ocular hemodynamics and cell viability (Toda and Nakanishi-Toda, 2007), being involved in vasodilation, neurotransmission and host cell defense (Christopherson and Bredt, 1997). However, NO produced at high concentrations by iNOS, leads to tissue injury, neurodegeneration, cell apoptosis (Christopherson and Bredt, 1997; Toda and Nakanishi-Toda, 2007), and inflammatory responses (Korhonen et al., 2005; Ricciardolo et al., 2006). Moreover, excess of NO can lead to the formation of peroxynitrite (Goureau et al., 1999; Ellis et al., 2002; Rosales et al., 2010; Hao et al., 2012), which is associated with DR and degenerative diseases.

Earlier studies have shown that the iNOS inhibitor, aminoguanidine, inhibited NO production, protein nitration, iNOS expression, vascular dysfunction, and retinal cell death in diabetic rats (Corbett et al., 1992; Du et al., 2002, 2004). Aminoguanidine also prevented the development of microvascular lesions (retinal microaneurysms, acellular capillaries, and pericyte ghosts) in type 1 diabetic dogs over a period of 5 years (Kern and Engerman, 2001). In streptozotocin (STZ)-induced diabetes in Long-Evans rats, aminoguanidine and 1400W (a more selective and potent iNOS inhibitor) restored light- and glial-evoked vasodilation in the retina, suggesting a role for iNOS in the loss of functional hyperemia due to aberrant glia-to-vessel signaling (Mishra and Newman, 2010). In a mouse model of oxygen-induced retinopathy, genetic deletion of iNOS or its blockade with 1400W increased physiological neovascularization and blunted the pathological intravitreal neovascularization (Sennlaub et al., 2001). In the same murine model, authors showed that iNOS is implicated in retinal degeneration through apoptosis (Sennlaub et al., 2002).

The kallikrein-kinin system is also involved in the development of DR (Wilkinson-Berka and Fletcher, 2004; Feener, 2010; Bhat et al., 2014). BK and KD are the agonists of the constitutive B2 receptor (B2R) while their metabolites produced by the CPM (Des-Arg^9^-BK and Des-Arg^10^-KD) are the preferred agonists of the inducible B1 receptor (B1R) (Regoli and Barabe, 1980; Regoli et al., 1998). Kinin receptors play an important role in the regulation of local blood flow, tissue edema, vasodilation, and leukocytes infiltration (Couture et al., 2001). B2R plays a vasodilator and vasoprotector role through activation of eNOS and prostanoids. B1R is undetectable in physiological conditions; it is induced in pathological conditions by pro-inflammatory cytokines and by oxidative stress associated with hyperglycemia (Leeb-Lundberg et al., 2005; Couture et al., 2014). The inducible B1R is implicated in complications of type 1 and type 2 diabetes (Couture et al., 2014), notably in DR. B1R was upregulated by an oxidative stress mechanism in the retina of STZ-diabetic rats (Abdouh et al., 2003, 2008; Pouliot et al., 2012) and its ocular blockade prevented the development of the pathogenesis of DR (Pouliot et al., 2012; Hachana et al., 2018). It was also shown that B1R is associated with the oxidative stress in the optic nerve and cortical visual area of 1 and 4 weeks STZ-diabetic rats (Catanzaro et al., 2017). It is worth noting that B1R activates iNOS through Gαi/ERK/MAPK signaling pathways in HEK293 cells (Brovkovych et al., 2011) and B1R antagonism reverses iNOS overexpression in the retina and pancreas of STZ-diabetic rats (Pouliot et al., 2012; Tidjane et al., 2016). These studies suggested that B1R is located upstream to iNOS activation and expression in diabetes.

To better understand the B1R-iNOS relationship in DR, we investigated the expression and distribution of B1R and iNOS in 2 weeks STZ-diabetic retinae to enable comparison with previous studies using B1R antagonists (Pouliot et al., 2012; Hachana et al., 2018). The impact of the iNOS inhibitor 1400W was determined on the expression of inflammatory mediators, notably iNOS, kallikrein-kinin system molecules, including carboxypeptidase M (CPM) which controls the activation of B1R, and VEGF which is involved in many aspects of vascular damage (Hoeben et al., 2004). The effect of 1400W on diabetes-induced nitrosative stress and retinal vascular permeability and on vascular hyperpermeability induced by a B1R agonist was also evaluated. This study highlights a partnership between B1R and iNOS in DR.

## Materials and Methods

### Model of STZ-Diabetic Rat

All experimental methods and animal care procedures were approved by the animal care committee of the Université de Montréal (Protocols 15-063, 16-059, and 17-057), in accordance with the Canadian Council on Animal Care. Animal studies are reported in compliance with the ARRIVE guidelines (Kilkenny et al., 2010; McGrath and Lilley, 2015). Six-week-old male Wistar rats weighing between 200 and 225 g were purchased from Charles River Laboratories (St-Constant, QC, Canada) and housed two per cage in a room under standard conditions (22.5°C and 42.5% humidity, on a 12 h/12 h light-dark cycle), with a standard chow diet (Charles River Rodent) and water provided *ad libitum*. Diabetes was induced by a single intraperitoneal injection of Streptozotocin (STZ, Zanosar 65 mg.kg^-1^, Cayman Chemical, Ann Arbor, MI, United States). Age-matched control rats were injected with vehicle used to solubilize STZ; 0.1M citrate buffer pH 5.

Glucose concentrations and body weight were recorded every 3 days and on the day of the experiments. Diabetes was confirmed by the measurement of a blood glucose concentration greater than 20 mmol.L^-1^ with a commercial blood glucose analyzer (Accusoft; Roche Diagnostics, Laval, QC, Canada) (Pouliot et al., 2012).

### Topical Ocular Treatment With the iNOS Selective Inhibitor 1400W

One week after diabetes induction, rats were treated twice a day (8 AM and 5 PM) with one eye drop application of the selective iNOS inhibitor 1400W for a 7-day period. N-(3-(aminomethyl) benzyl)acetamidine (1400W, Cayman Chemical, Ann Arbor, United States) is an extremely slowly reversible inhibitor for human and rat iNOS with a 5000-fold selectivity for iNOS *versus* eNOS and nNOS (Garvey et al., 1997). In a previous study, we reported that 1400W can prevent the deleterious effects of B1R in insulin resistance and peripheral inflammation by blocking the formation of peroxynitrite (Haddad and Couture, 2016). In a model of ischemic retinopathy, 1400W also inhibited the pathological neovascularization (Sennlaub et al., 2001, 2002). For our study, fresh solution (15 mg.mL^-1^) was prepared daily with sterile saline 0.9% and filtrated (0.20 μm mesh). This dosage was chosen based on a pilot dose-response study where 10 μL of 5 mg.mL^-1^ failed to affect the increase of vascular permeability in STZ-retina. The latter inflammatory response was slightly decreased with 10 μL of 10 mg.mL^-1^, and completely reversed with 10 μL of 15 mg.mL^-1^. In the present study, 10 μL of the 15 mg.mL^-1^ solution (i.e., 150 μg in 10 μl) was administered twice a day on the surface of the eye using a pipette. To make sure that the drop effectively remained on the surface of the eye, animals were hand-restrained for 60 s. The presence of ocular irritation such as redness, porphyrin secretion or corneal opacity was verified daily.

### Intravitreal Injection of the B1R Agonist R-838

To assess the contribution of iNOS in B1R-induced vascular permeability, twenty rats were randomly divided into two control groups and two STZ-diabetic groups of 5 rats per group. Control groups and STZ-diabetic groups were treated either with the vehicle or with 1400W by eye-drops. Pupils of isoflurane anesthetized rats were dilated with 0.5% tropicamide to allow intravitreal injections on day 10 and 12. Under a dissecting microscope, a small incision of 1mm was done behind the limbus using a 30-gauge needle to facilitate the intravitreal injections. A sterile solution of the B1R agonist R-838 synthesized at the Research Institute of Biotechnology, National Research Council of Canada (Montreal, QC, Canada) (100 ng in 5 μL of 0.9% saline) was injected into the vitreous through the incision using a glass micropipette sealed at the tip of the Hamilton microsyringe (10 μL) (Hamilton, Reno, NV, United States) according to our previous study (Hachana et al., 2018). The contralateral eye received intravitreal injection of the vehicle (saline) for comparison. Retina samples were collected at day 15 to measure the vascular permeability using the Evans blue dye technique.

### Measurement of Retinal Vascular Permeability

Retinal vascular permeability was measured using Evans blue dye extravasation technique as previously described (Abdouh et al., 2008; Pouliot et al., 2012). Rats underwent general anesthesia by intraperitoneal injection of sodium pentobarbital (60 mg.kg^-1^). A catheter (Micro-Renathane, I.D. 0.040′′, O.D. 0.020′′, Braintree Scientific, Braintree, MA, United States) was inserted into the right femoral vein and Evans blue dye (45 mg.mL^-1^ in 0.9% saline) (Sigma-Aldrich, Oakville, ON, Canada) was injected i.v. over 10 s. The rats were kept on a heating pad for 2 h to allow the dye to circulate, while the body temperature was monitored at every 10 min. Thereafter, the rats were infused with a 25 mL of sterile saline 0.9% through the left heart ventricle, to wash out intravascular dye. Both eyes were then enucleated, and the retinae were dissected out and immediately weighed. Evans blue dye was extracted by incubating each retina in 1 mL formamide (Sigma-Aldrich, Oakville, ON, Canada) for 18 h at 70–75°C. The fluorescence of Evans blue was measured using a spectrofluorometer (Spex 1681 0.22 m, Horiba JobinYvon Inc, Edison, NJ, United States) at 620 nm (excitation) and 680 nm (emission). Based on a standard curve, data were calculated in μg of Evans blue and then expressed per g of fresh tissue.

### Measurement of Retinal Inflammatory Mediators by Quantitative RT-PCR

As previously described (Pouliot et al., 2012), rats were anesthetized with sodium pentobarbital (60 mg.kg^-1^, i.p.) and the retinae were dissected out and put in RNA*later* stabilization reagent (QIAGEN, Valencia, CA, United States). Total RNA was extracted from retinae using a commercial kit (QIAGEN, Valencia, CA, United States). First-strand cDNA synthesized from 400 ng total RNA with random hexamer primers was used as template for each reaction with the QuantiTect Rev Transcription Kit (QIAGEN). SYBR Green-based real-time quantitative PCR using Mx3000p device for signal detection (Stratagene, La Jolla, CA, United States) was performed as previously described (Abdouh et al., 2008; Pouliot et al., 2012). PCR was performed in SYBR Green Master mix (QIAGEN) with 300 nM of each primer. The primer pairs designed by Vector NTI software are shown in Table 1. For standardization and quantification, rat 18S was amplified simultaneously. PCR conditions were as follows: 95°C for 15 min, followed by 46 cycles at 94°C for 15 s, 60°C for 30 s, and 72°C for 30 s. The cycle threshold (Ct) value represents the cycle number at which a fluorescent signal rises statistically above background. The relative quantification of gene expression was analyzed by the 2^-ΔΔCt^ method.

**Table 1:** List of primers designed by vector NTI software and used in qRT-PCR analysis.

Gene			Sequence		Position	GenBank accession no
B1R	Forward	5′	GCAGCGCTTAACCATAGCGGAAAT	3′	367–390	NM_030851
	Reverse	5′	CCAGTTGAAACGGTTCCCGATGTT	3′	454–431	
B2R	Forward	5′	AGGTGCTGAGGAACAACGAGATGA	3′	882–905	NM_173100
	Reverse	5′	TCCAGGAAGGTGCTGATCTGGAAA	3′	990–967	
iNOS	Forward	5′	TGATCTTGTGCTGGAGGTGACCAT	3′	1150–1173	NM_012611
	Reverse	5′	TGTAGCGCTGTGTGTCACAGAAGT	3′	1349–1326	
VEGF-A	Forward	5′	TCACCAAAGCCAGCACATAGGAGA	3′	1219–1242	BC168708
	Reverse	5′	TTACACGTCTGCGGATCTTGGACA	3′	1371–1348	
VEGF-R2	Forward	5′	AGTGGCTAAGGGCATGGAGTTCTT	3′	3269–3292	U93306
	Reverse	5′	GGGCCAAGCCAAAGTCACAGATTT	3′	3387–3364	
IL-1β	Forward	5′	TGTCACTCATTGTGGCTGTGGAGA	3′	247–270	NM_031512
	Reverse	5′	TGGGAACATCACACACTAGCAGGT	3′	411–388	
TNF-α	Forward	5′	ACGGAAAGCATGATCCGAGATGTG	3′	151–174	NM_012675
	Reverse	5′	TTGGGAACTTCTCCTCCTTGTTGG	3′	340–317	
18S	Forward	5′	TCAACTTTCGATGGTAGTCGCCGT	3′	363–386	X01117
	Reverse	5′	TCCTTGGATGTGGTAGCCGTTTCT	3′	470–447	


### Measurement of Retinal Inflammatory Mediators by Western Blot

The measurement of protein expression of retinal inflammatory mediators was done using the Western Blot technique as previously described (Pouliot et al., 2011; Haddad and Couture, 2016). 40 μg of protein were loaded in each well of 10% SDS-PAGE and separated electrophoretically at 100 V for 125 min. Proteins were then transferred onto a nitrocellulose membrane (Bio-Rad, Montreal, QC, Canada) at 100 V for 1 h. After 3 sets of 5 min washing in phosphate-buffered saline (PBS), the membranes were incubated in a blocking solution with PBS-Tween 20 containing 5% of dehydrated skimmed milk at 4°C for 1 h. The membranes were then incubated with specific primary antibody in 5% skimmed milk overnight at 4°C. Dynein and β-actin were used as standard proteins. The membranes were washed again 3 × 10 min with PBS-Tween 20. The antibody-antigen complexes were detected by adding secondary antibody in 1% skimmed milk at room temperature for 1 h and protein bands were visualized by enhanced chemiluminescence improved for the western blot (Super-Signal^^®^^; Thermo Scientific, Rockford, IL, United States). Quantitative analysis of specific bands was performed by densitometry using the MCID-M1 system (Imaging Research, St. Catharines, ON, Canada).

As described previously (Haddad and Couture, 2016), the detection of BK receptor proteins was made with selective polyclonal rabbit antiserum for B1R (1:1000), and B2R (1:1000) (Biotechnology Research Institute, Montreal, QC, Canada). Specificity of both antisera was determined in B1 and B2 receptor knockout (KO) mice and by siRNA technology (Dias et al., 2010; Lacoste et al., 2013; Hachana et al., 2018). The other primary antibodies were as follows: iNOS (NOS2) (1:1500, rabbit, SC-650), IL-1β (1:500, rabbit, SC-7884), CPM (1:500, rabbit, SC-98698), dynein (1:5000, mouse, SC-13524), β-actin (1:5000, mouse, SC-47778), (SC: Santa Cruz Biotechnology, CA, United States), nitrotyrosine (1:1500 mouse, 1A6-05233; Millipores, Billerica, MA, United States), and VEGF_164_ (1:1000, goat, AF564; R&D systems, Minneapolis, MN, United States). Secondary antibodies were horseradish peroxidase (HRP)-linked with: goat anti-mouse (SC-2005) or goat anti-rabbit (SC-2004) or donkey anti-goat (SC-2020) and HRP-linked used at dilution of 1:25000 (for B1R and B2R), 1:4000 (for dynein, β-actin, IL-1β, iNOS, VEGF, and CPM) and 1:3000 (nitrotyrosine). The nitration of protein tyrosine residues generating 3-nitrotyrosine has been used as a footprint for the formation of peroxynitrite *in-vivo* (Greenacre and Ischiropoulos, 2001).

### Immunofluorescence Staining

At day 15, rats were anesthetized with sodium pentobarbital (60 mg.kg^-1^, i.p.) and then perfused with a paraformaldehyde solution (4% PFA). Dissected retinae were then post-fixed in 4% PFA for 2 h and kept in 70% alcohol overnight at room temperature for a paraffin embedding. The retinae were then cut into 10-μm-thick sections and placed onto glass slides and kept at 4°C till the day of experiment. For proteins detection, the sections were deparaffinized prior to the immunofluorescence experiment. First, glass slides were incubated in sodium citrate buffer at 95°C for 45 min. Sections were then allowed to cool down for 20 min. Sections were then washed 3 times (5 min each) with 0.1 M PBS buffer (pH 7.4) and incubated for 1 h at room temperature in blocking buffer (PBS containing 10% donkey serum and 0.25% triton X-100). Sections were left incubated overnight at room temperature with the blocking buffer containing primary antibodies: polyclonal rabbit antiserum to rat B1R (1:200), mouse monoclonal anti-iNOS (1:200, mouse, ab49999; Abcam, Cambridge, United States). The following day, slides were washed 3 × 5 min in PBS 0.1M and then incubated for 2 h at room temperature with Alexa Fluor 488 donkey secondary anti-rabbit IgG (1:200, A21206, Invitrogen) to visualize B1R, with Alexa Fluor 555 donkey anti-mouse IgG (1:200, A31570, Invitrogen) to visualize iNOS. At the end, the slides were washed and mounted using glycerol solution. Images were obtained with a confocal microscope Zeiss-LSM800 equipped with an argon laser (Carl Zeiss, Jena, Germany) and transferred to a computer and analyzed using NIH ImageJ 1.36b Software (NIH, Bethesda, MD, United States). Images were obtained at 20x and 40x objective. Semi-quantification of immunofluorescence staining intensity was made on 5 randomly selected surface areas of each retina from four STZ-diabetic rats, four controls and four STZ-diabetic treated rats. Background intensity (gray intensity) was subtracted from each individual value.

### Statistical Analysis

Statistical analysis was performed using Prism^TM^ version 5.0 (GraphPad Software Inc., La Jolla, CA, United States). Results are expressed as the mean ± SEM and *n* represents the number of rats used in each experiment. One-way ANOVA followed by Tukey’s *post hoc* test was used for all the measurements. Results were considered significant at a value of *P* < 0.05.

## Results

### Physiological Parameters

At the time of sacrifice, blood glucose concentration was significantly higher in STZ-diabetic groups compared with control rats, but no statistical difference was seen between non-treated and treated STZ-diabetic rats with R-838 and/or 1400W (Table 2). Body weight of STZ-diabetic rats was significantly lower than the one of age-matched control rats. Administration of R-838 and/or 1400W had no effect on body weight in both control and STZ-diabetic rats (Table 2). Of note, no symptoms of ocular irritation (redness or corneal opacity) or the presence of porphyrin secretion around the eyes were seen during the whole period of treatment.

**Table 2:** Effects of diabetes, B1R agonist (R-838) and iNOS inhibitor (1400W) on body weight and glycaemia.

	Body	Glycaemia
Groups	weight (g)	(mmol.L^-1^)
Control (*n* = 12)	382 ± 5	5.0 ± 0.1
Control + R-838/vehicle (*n* = 5)	373 ± 7	5.1 ± 0.1
Control + R-838/vehicle + 1400W (*n* = 5)	379 ± 9	5.2 ± 0.1
STZ (*n* = 12)	314 ± 9^∗^	27.8 ± 0.7^∗^
STZ + 1400W (*n* = 12)	300 ± 10^∗^	27.7 ± 0.7^∗^
STZ + R-838/vehicle (*n* = 5)	291 ± 6^∗^	29.2 ± 1.2^∗^
STZ + R-838/vehicle + 1400W (*n* = 5)	292 ± 7^∗^	28.8 ± 0.9^∗^


*Values represent the mean ± S.E.M of five to twelve rats per group (as indicated by n). R-838 was administered in one eye and the effect compared to the opposite eye treated with the vehicle. Statistical significance was determined with one-way ANOVA followed by the Tukey Test. ^∗^*P* < 0.05 significantly different from the corresponding control. STZ, streptozotocin-treated rats.*

### Effect of iNOS Inhibition on Protein and mRNA Levels of Immunomodulators in Diabetic Retina

**FIGURE 1:**
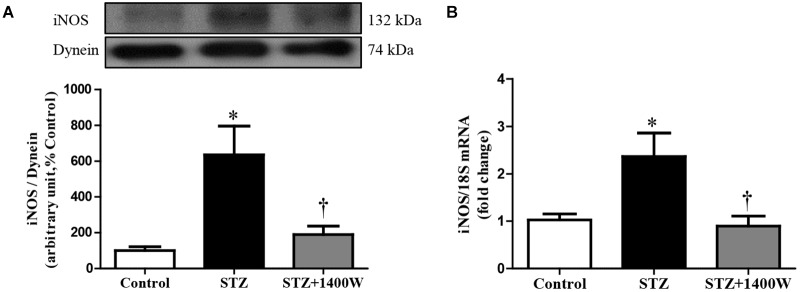
Effect of STZ-induced diabetes and eye-drops 1400W treatment on protein **(A)** and mRNA **(B)** expression of iNOS in the retina. Data are mean ± SEM of values obtained from five rats in each group. ^∗^*P* < 0.05 STZ-diabetic compared with control; ^†^*P* < 0.05 STZ + 1400W compared with STZ.

**FIGURE 2:**
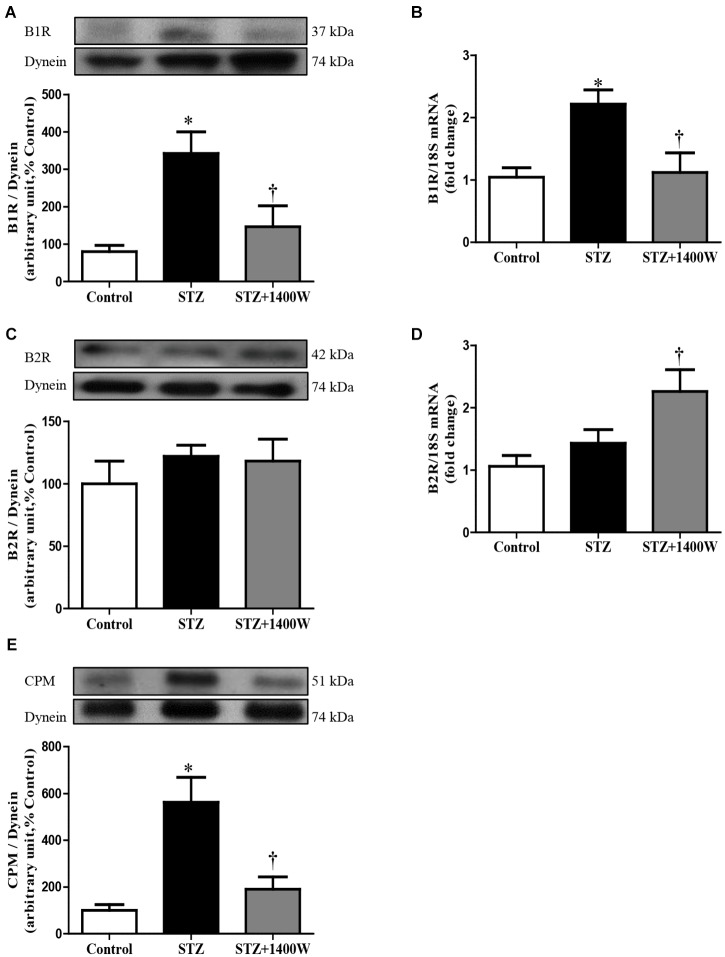
Effect of STZ-induced diabetes and eye-drops 1400W treatment on the expression of kinin B1R **(A,B)**, B2R **(C,D)**, and CPM **(E)** at protein **(A,C,E)** and mRNA **(B,D)** levels in the retina. Data are mean ± SEM of values obtained from five rats per group or six rats in CPM group. ^∗^*P* < 0.05 STZ-diabetic compared with control; ^†^*P* < 0.05 STZ + 1400W compared with STZ.

An increase of iNOS expression was seen concomitantly with an elevated expression of B1R and CPM in STZ-diabetic retina. In comparison to control retina, iNOS expression was increased by six-fold at protein level, and by 2.5-fold at mRNA level in diabetic retinae (Figure 1A,B). B1R expression was also increased by ≈3.5-fold at protein level and 2.2-fold at mRNA level in STZ-diabetic retina (Figure 2A,B). CPM protein expression was increased by ≈ six-fold in STZ-diabetic retina (Figure 2E). The increased expression of iNOS, B1R and CPM were reversed to control levels by the treatment with 1400W, providing evidence that iNOS inhibition reversed not only B1R expression but also the biosynthesis of B1R agonists by CPM. This occurs without significant changes on B2R protein expression, yet 1400W significantly increased B2R mRNA level in STZ-retina (Figure 2C,D).

**FIGURE 3:**
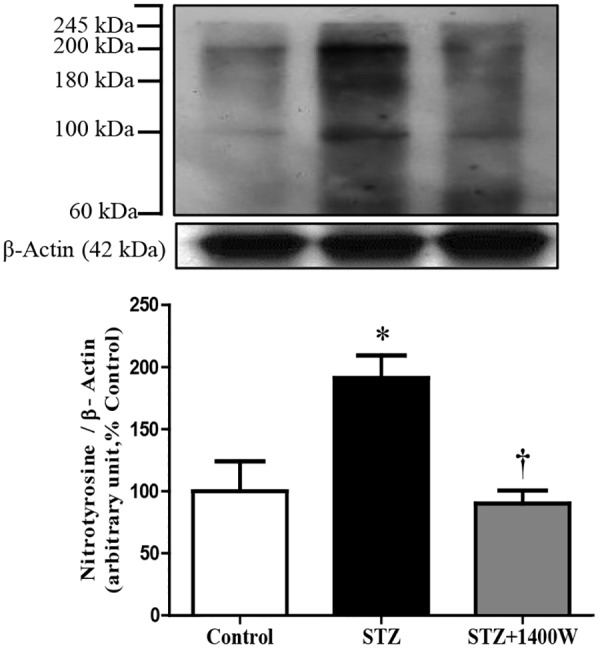
Effect of STZ-induced diabetes and eye-drops 1400W treatment on nitrotyrosine expression in the retina. Data are mean ± SEM of values from 3 bands (100, 180, and 200 kDa) obtained from five rats per group. Background intensity was subtracted from each individual value.^∗^*P* < 0.05 STZ-diabetic compared with control; ^†^*P* < 0.05 STZ + 1400W compared with STZ.

Western blot analysis revealed that the intensity of several nitrosylated protein bands, notably at 200, 180, and 100 kDa, increased in the retina of STZ-diabetic rats compared to control (Figure 3). The eye-drops application of 1400W reversed the nitration reaction to control values, suggesting that iNOS is involved in protein nitration. This possibility is supported by the absence of nitrotyrosine residues in the retina of knockout iNOS mice in comparison with wild-type mice in a model of ischemic retina (Sennlaub et al., 2001).

**FIGURE 4:**
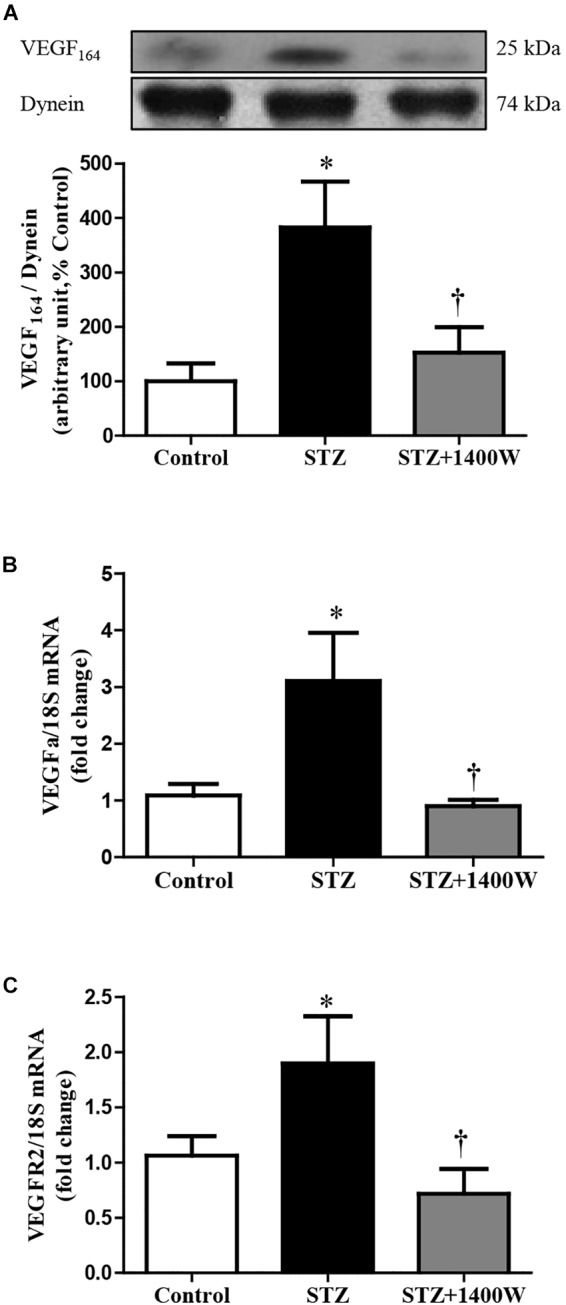
Effect of STZ-induced diabetes and eye-drops 1400W treatment on protein expression of VEGF_164_ **(A)**, mRNA levels of VEGF-A **(B)**, and VEGF-R2 **(C)** in the retina. Data are mean ± SEM of values obtained from five to six rats in each group. ^∗^*P* < 0.05 STZ-diabetic compared with control. ^†^*P* < 0.05 STZ + 1400W compared with STZ.

**FIGURE 5:**
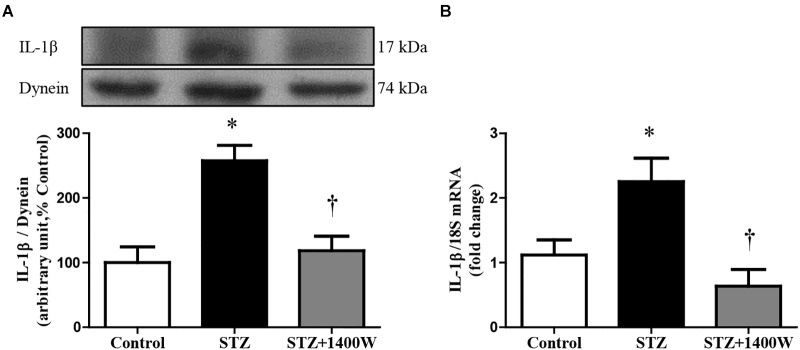
Effect of STZ-induced diabetes and eye-drops 1400W treatment on protein **(A)** and mRNA **(B)** expression of IL-1β in the retina. Data are mean ± SEM of values obtained from five rats in each group. ^∗^*P* < 0.05 STZ-diabetic compared with control; ^†^*P* < 0.05 STZ + 1400W compared with STZ.

VEGF-A plays a key role in increasing vascular permeability and promoting the angiogenesis in DR. Thus, intravitreal injections of anti-VEGF are one of the treatments used to stop DR progression. The protein expression of VEGF_164,_ which is one isoform of VEGF-A, was increased by four-fold while the mRNA level of VEGF-A was increased by 3-fold in diabetic retina (Figure 4A,B). The enhanced expression of VEGF-A was reversed by the 1-week treatment with 1400W. Likewise, VEGF-R2 expression was significantly increased at mRNA level in STZ-diabetic retina and normalized by treatment with 1400W (Figure 4C). Similarly, the expression of IL-1β was increased by 2.5-fold at protein and mRNA levels in STZ-retina, and this was completely reversed by 1400W treatment (Figure 5A,B). This cytokine is known to enhance the expression of B1R through NF-κB. The mRNA expression of TNF-α, another cytokine involved in the induction of B1R (Leeb-Lundberg et al., 2005), was also significantly enhanced in diabetic retina and normalized by the treatment with 1400W (fold change of control: 1.0 ± 0.2; STZ: 4.7 ± 0.9, *P* < 0.05; STZ + 1400W: 2.0 ± 0.3, *P* < 0.05, *n* = 5).

### Effect of iNOS Inhibition on B1R and iNOS Immunoreactivity in the Diabetic Retina

**FIGURE 6:**
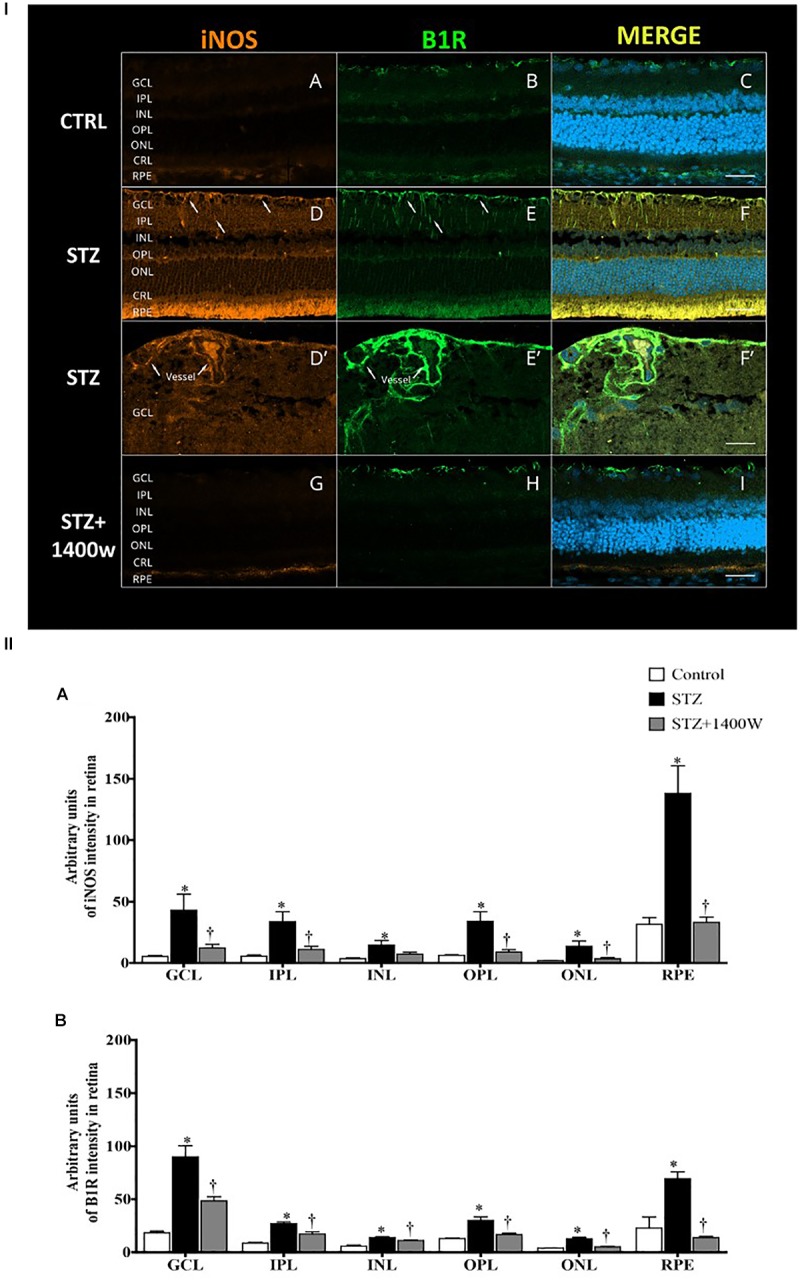
**(I)** Microphotographs of immunolocalization of iNOS **(**orange, **A**,**D**,**D’**,**G)** and B1R **(**green, **B**,**E**,**E’**,**H)** in the retina of control (CTRL) and STZ-diabetic rats treated by eye-drops with vehicle or 1400W. Representative microphotographs of merge immunolabeling are shown **(C**,**F**,**F’**,**I)**. All sections were counter-stained for DAPI (blue, shown on merged images only), which labels cell nuclei. Arrows show the presence of iNOS and B1R in the same areas of GCL and IPL **(D**,**E)**, notably on (blood vessels **(D’**,**E’)**. Both proteins were colocalized in the STZ-retina **(F**,**F’)** but not in control **(C)** or STZ-retina treated with 1400W **(I)**. Images were obtained at 20× **(A–F**, **G–I)** and 40× **(D’–F’)** objective. Scale bar: 40 μm **(A–F**,**G–I)** and 20 μm **(D’–F’)**. CRL, cones and rods layer; GCL, ganglion cell layer; INL, inner nuclear layer; IPL, inner plexiform layer; ONL, outer nuclear layer; OPL, outer plexiform layer; RPE, retinal pigmented epithelium. **(II)** Quantification of the fluorescent intensity is depicted for iNOS **(A)** and B1R **(B)** in the different layers of the retina. Data are mean ± SEM of values obtained from four rats per group and four sections per animal. ^∗^*P* < 0.05 STZ-diabetic compared with control; ^†^*P* < 0.05 STZ + 1400W compared with STZ.)

The immunofluorescence staining confirmed the enhanced levels of iNOS and B1R in the STZ-diabetic retina. In contrast, iNOS and B1R immunostaining was slightly detectable in control retina and no colocalization was evidenced (Figure 6I A–C). STZ-induced diabetes increased strongly the expression of iNOS and B1R in all the retina layers (Figure 6I D,E). Both markers were mainly colocalized in the GCL, inner retina (INL and IPL) and the retinal pigmented layer (Figure 6I F). The latter forms the outer blood-retinal barrier. iNOS and B1R are also colocalized on blood vessels in the GCL (Figure 6I D’–F’). iNOS inhibition with 1400W decreased the high staining and colocalization of B1R and iNOS induced by diabetes (Figure 6I G–I), suggesting an implication of iNOS in the positive feedback regulation of B1R expression. Semi-quantitative values of iNOS and B1R intensity of immunofluorescence staining in control and STZ-diabetic retina without and with 1400W treatment are given in Figure 6II A,B.

### Effect of iNOS Inhibition and B1R Activation on Retinal Vascular Permeability

**FIGURE 7:**
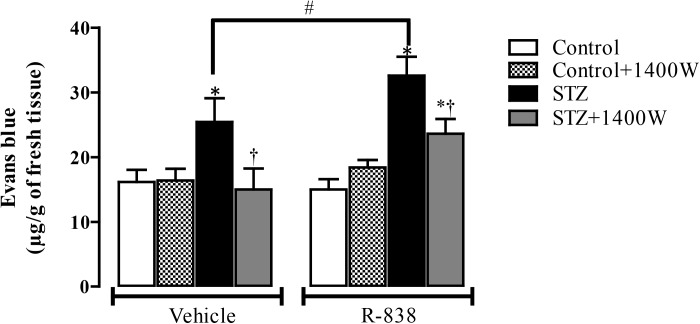
Effect of STZ-induced diabetes, eye-drops treatment with 1400W and intravitreal B1R agonist (R-838) on retinal vascular permeability. Data are mean ± SEM obtained from 12 rats per group (vehicle) and 5 rats per group (R-838 and control + 1400W). ^∗^*P* < 0.05 STZ-diabetic compared with control; ^†^*P* < 0.05 STZ + 1400W compared with STZ; ^#^*P* < 0.05 between STZ treated with vehicle and R-838.

Following the upregulation of B1R and iNOS in the STZ-diabetic retina, we determined whether B1R was functional and could interact with iNOS in an inflammatory response such as the breakage of blood-retinal barrier. The 1-week treatment with 1400W reversed the STZ-induced increase in vascular permeability and reduced significantly the response to the B1R agonist in diabetic retina (Figure 7). The iNOS inhibitor had no impact in control rats treated with vehicle or R-838, which do not overexpress B1R. This provides evidence that iNOS activation mediates the B1R retinal vascular hyperpermeability in diabetes.

## Discussion

**FIGURE 8:**
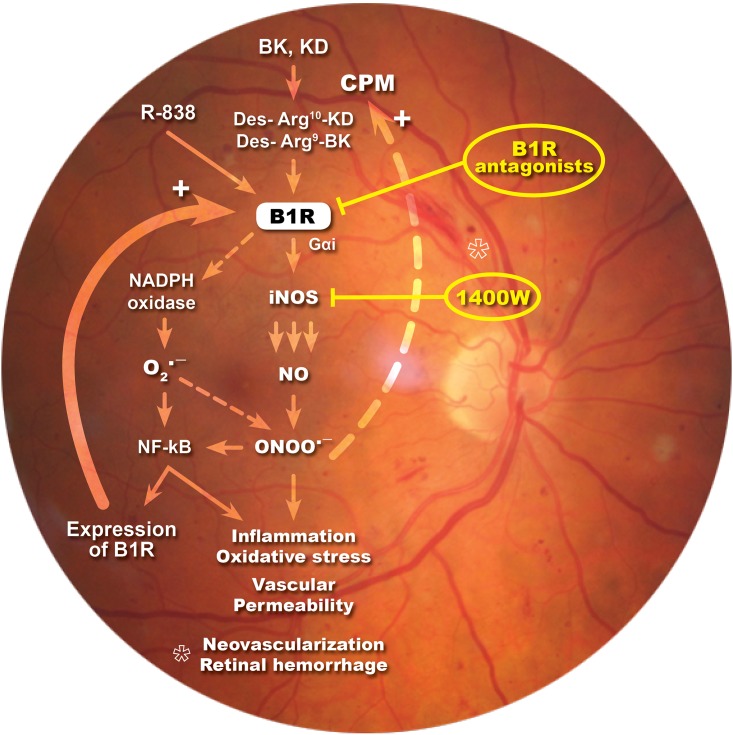
Schematic diagram showing how B1R and iNOS can partake in a mutual auto-induction and amplification loop to enhance nitrogen species formation and inflammation in diabetic retina. B1R agonists enhance the formation of peroxynitrite (ONOO^-^) through the activation of iNOS and NADPH oxidase pathways. iNOS activation produces high amount of NO while NADPH oxidase activation leads to superoxide anion (O_2_^∙^-^^) formation that can rapidly react with NO to form ONOO^-^. Peroxynitrite formation induces an inflammatory response, enhances the oxidative stress and vascular permeability, neovascularization and retinal hemorrhage. Both O_2_^∙^-^^ and ONOO^-^ are known to activate the transcriptional nuclear factor, NF-κB, which is involved in the transcription of several inflammatory genes, notably iNOS, B1R, and CPM. iNOS inhibitor (1400W) and B1R antagonists prevent this vicious cycle and the pro-inflammatory effect of B1R.

In the present study, we showed that iNOS plays an important role in the development of vascular damage linked to DR and in B1R-induced vascular permeability. We found that eye-drops application of the selective iNOS inhibitor, 1400W, reversed inflammatory response, nitrosative stress and vascular hyperpermeability in the retina of STZ-diabetic rats. Importantly, this study provides the first evidence that B1R is colocalized with iNOS throughout the retina and that B1R activates iNOS to enhance vascular permeability in this model of DR. A positive loop is suggested between iNOS and B1R to amplify and perpetuate the retinal inflammatory process. This is keeping with earlier data showing that B1R antagonism (Pouliot et al., 2012; Hachana et al., 2018) had similar beneficial effects than iNOS inhibition in this model of DR (Figure 8).

### iNOS Implication in Diabetic Retinopathy

The increase of the oxidative stress is considered as an important factor in the pathophysiology of DR. Chronic hyperglycemia induces oxidative stress, which can consequently damage proteins, lipids, and DNA (Stadler, 2011). Retinal vascular oxidative stress persisted even after glucose level normalization (Ihnat et al., 2007). Increased retinal expression of iNOS is known to be a key factor responsible for diabetes-induced retinal inflammation (Abu El-Asrar et al., 2004; Kern, 2007). Our results with 1400W are in agreement with the implication of iNOS through the oxidative stress in the onset of DR (Rosales et al., 2010; Silva et al., 2010).

Unlike the constitutive NOS, iNOS is activated under hypoxic and inflammatory conditions leading thus to excessive amounts of NO, which might contribute to the pathogenesis of DR (Abu El-Asrar et al., 2001; Rosales et al., 2010; Silva et al., 2010). Therefore, our approach was to selectively block iNOS, and leave intact nNOS and eNOS which play important physiological functions such as vasodilation and blood flow regulation. NO synthesized from iNOS promotes peroxynitrite formation, which is implicated in retinal endothelial and neuronal cell death, retinal neurodegeneration, and blood-retinal barrier breakdown in models of experimental diabetes (El-Remessy et al., 2003; el-Remessy et al., 2005; Zheng et al., 2007; Ali et al., 2008). In addition, peroxynitrite and the oxidative stress can activate the transcriptional nuclear factor NF-κB, which is known to increase the transcription of several inflammatory cytokines such as IL-1β and TNF-α, iNOS, and B1R (Leeb-Lundberg et al., 2005; Pall, 2013). Here, we found that elevated expression of iNOS associated with diabetes is accompanied by an elevated expression of nitrosylated proteins that is reversed by iNOS inhibition. This agrees with diabetic mice lacking iNOS which are protected from the diabetes-induced nitration of retinal proteins (Zheng et al., 2007). Consistently, nitrotyrosine immunolabeling was reported in the inner retina and retinal vasculature of diabetic rats (Du et al., 2002; Liu et al., 2013) and in diabetic human retina (Abu El-Asrar et al., 2004).

In a model of type 2 diabetes, iNOS was found expressed in the GCL, the INL, and at the ONL (Carmo et al., 2000). Also, a cytoplasm-associated immunoreactivity of iNOS was seen in ganglion cells, INL, glial cells, and vessels of diabetic human retinae (Abu El-Asrar et al., 2004). Recently, we reported B1R expression in retinal ganglion cells, INL, and Müller cells in STZ-diabetic retina (Hachana et al., 2018). In the present study, we show an increased expression of B1R in diabetic retina accompanied by an increased expression of iNOS in the same regions. B1R and iNOS immunoreactivities were colocalized in the GCL, on blood vessels, the INL, and RPE. This colocalization may suggest interaction between both proteins.

### B1R Implication in Diabetic Retinopathy

We have previously shown that B1R is implicated in the very early stage of DR (Abdouh et al., 2003, 2008; Pouliot et al., 2011, 2012; Hachana et al., 2018) and that B1R exerts potent effects on retinal vascular hyperpermeability. Herein we show again an increased retinal B1R expression at 2 weeks of diabetes. We found that the impact of B1R on retinal vascular permeability in diabetic rats is stronger when B1R is stimulated by its agonist (R-838). It has been previously shown that B1R activates NADPH oxidase leading to increased production of superoxide anion in STZ-diabetic vessels through PKCβ1/2 (Haddad and Couture, 2017). Importantly, B1R enhanced iNOS expression in diabetic pancreas, adipose tissue, vessels and retina (Dias and Couture, 2012a,b; Pouliot et al., 2012; Tidjane et al., 2016), which is keeping with the activation of iNOS by B1R through Gαi/ERK/MAPK signaling (Brovkovych et al., 2011). Hence the interaction between B1R, iNOS and NADPH oxidase represents a reasonable mechanism for the generation of nitrosylated proteins (Figure 3) through peroxynitrite formation. The latter is the result of a combination of NO (produced by iNOS) and superoxide anion derived from NADPH oxidase (Figure 8). This is expected to amplify the pro-inflammatory effects and expression of B1R and iNOS through a positive feedback loop as shown in other tissues of diabetic rats (Haddad and Couture, 2016). It seems that peroxynitrite can also exert a positive feedback loop to enhance the expression of CPM, yielding endogenous agonists to activate B1R. These results are in line with our previous study where we showed that enhanced iNOS expression in different organs of STZ-diabetic rats increases CPM expression through peroxynitrite formation (Haddad and Couture, 2016). Collectively, the present data suggest a strong interaction between B1R and iNOS, which is further supported by the immunofluorescence study highlighting strong co-expression of iNOS and B1R.

## Conclusion

This study shows that iNOS and B1R play a critical role in the early stage of DR. B1R and iNOS appear to partake in a mutual auto-induction and amplification loop to enhance nitrogen species formation and inflammation in diabetic retina. Hence, targeting the B1R-iNOS axis can represent a promising and non-invasive therapeutic approach in DR. However, further studies are needed to confirm the concentration and any potential side effect of 1400W reaching to the retinal, corneal and choroidal compartments.

## Data Availability

The datasets generated for this study are available on request to the corresponding author.

## Author Contributions

RO, EV, and RC conceived and designed the experiments. RO performed the experiments, analyzed the data, and drafted the manuscript. RC and EV supervised the study, edited, and wrote the final version of the manuscript. All authors approved the final manuscript.

## Conflict of Interest Statement

The authors declare that the research was conducted in the absence of any commercial or financial relationships that could be construed as a potential conflict of interest.

## Abbreviations

1400W: N-[3-(aminomethyl)benzyl]acetamidine
BK: bradykinin
B1R: bradykinin type 1 receptor
B2R: bradykinin type 2 receptor
CRL: Cones and rods layer
CPM: carboxypeptidase M
DR: diabetic retinopathy
GCL: ganglion cell layer
IL-1β: interleukin-1β
INL: inner nuclear layer
iNOS: inducible nitric oxide synthase
IPL: inner plexiform layer
KD: kallidin
NO: nitric oxide
ONL: outer nuclear layer
OPL: outer plexiform layer
RPE: retinal pigmented epithelium
R-838: Sar-[D-Phe^8^]-des-Arg^9^-BK
STZ: streptozotocin
TNF-α: tumor necrosis factor-α
VEGF-A: vascular endothelial growth factor
VEGF-R2: receptor type 2 of VEGF
